# 

**Published:** 1846-07

**Authors:** 


*To Readers and Correspondents.*—Communications have been received from Dr. CONGER, Niagara Falls, and Dr. TREAT, of this city, which will appear in our next. We are happy to inform our readers that Prof. HAMILTON will continue his "Notes of an European Tour." His future letters will relate to medical matters in London, Dublin, Edinburg, &c. We beg to acknowledge the receipt of a letter from the Editor of the Dublin Quarterly Journal of Medical Science, and shall be happy to accept his complimentary invitation to exchange. Our Journal will be forwarded as suggested. The numbers sent to our address have not yet come to hand. Our usual exchanges have been regularly received.

☞ We have continued our second volume to the subscribers to the first volume. We would respectfully ask them to remit the subscription for the current year. Should any decline to have it continued, they are requested to return the Nos. sent. We hope, however, that none will be disposed to comply with the latter request, but that all will prefer to continue the work, and so signify by *remitting without delay.*

